# Supplementary material for: Species-specific retention vs. recovery of coral thermal tolerance following nursery propagation
Source: Commun Biol. 2025 Aug 28;8:1294. doi: 10.1038/s42003-025-08657-w (PMC12394514; doi:10.1038/s42003-025-08657-w)
Supplement: Supplementary file 2 — Supplementary Material [file 42003_2025_8657_MOESM2_ESM.pdf]

Electronic Supplementary Material accompanying the article:

Species-specific retention vs. recovery of coral thermal tolerance following nursery propagation

Sebastian Szereday[1]* | Kok Lynn Chew[1] | Christian R. Voolstra[2]

[1]Non-profit enterprise for coral reef research and restoration, Coralku Solutions, Kuala Lumpur, Malaysia.

[2]Department of Biology, University of Konstanz, Konstanz, Germany.

*** Correspondence:**
Sebastian Szereday
sebastian@coralku.org

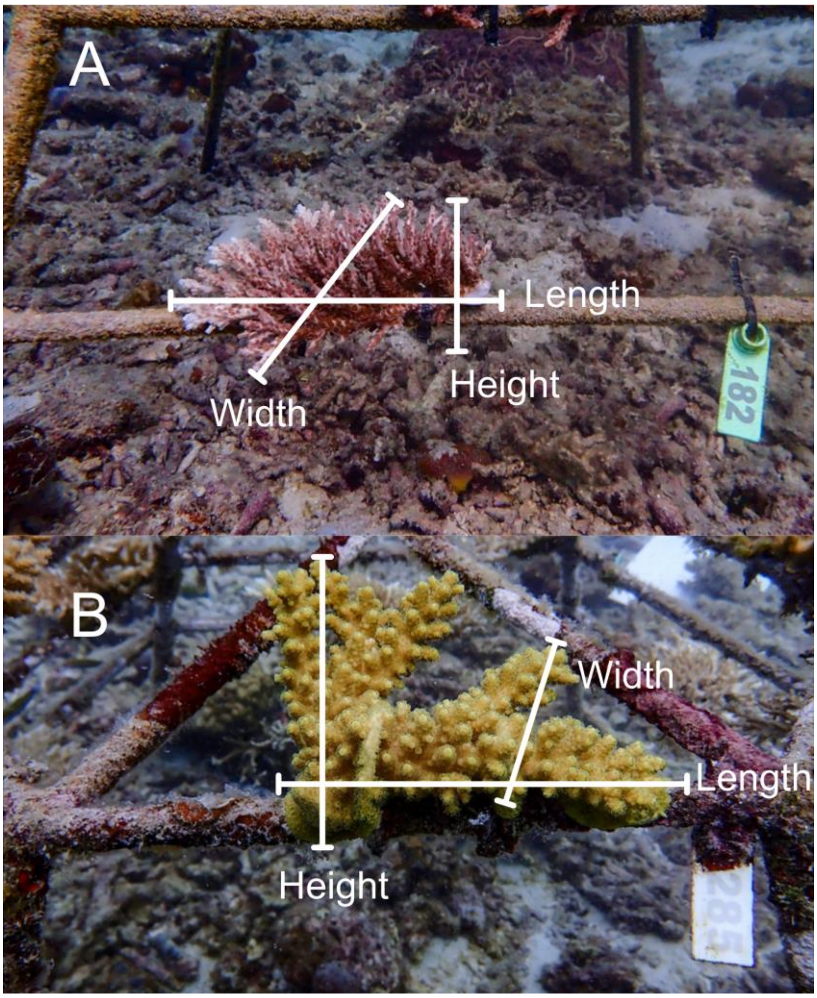

Supplementary Figure 1. Example of coral size measurements for (A) *Acropora cytherea*, and (B) *Acropora florida*.

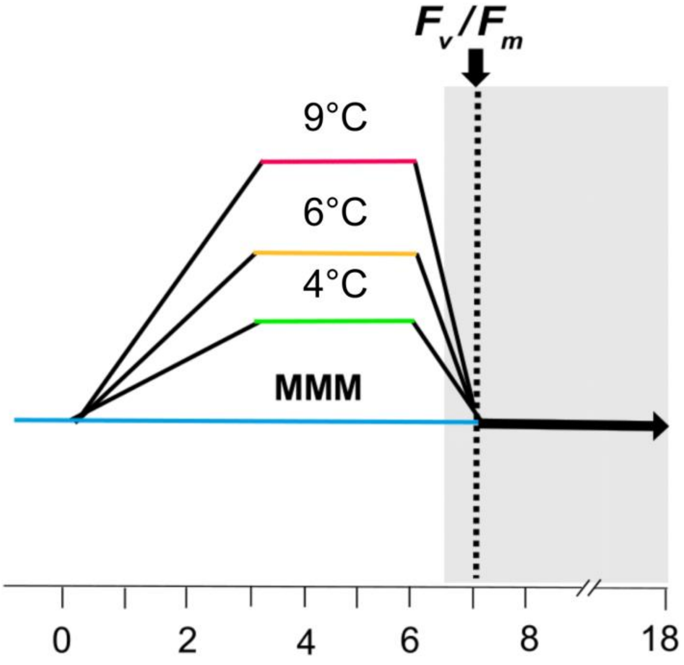

Supplementary Figure 2. Acute heat stress assay profiles of Coral Bleaching Automated Stress System (CBASS) with respective 3h maximum temperature treatments at 30.5 °C (baseline = maximum monthly mean MMM), 34.5 °C, 36.5 °C, and 38.5 °C. Maximum photosynthetic efficiency of PSII (Fv/Fm) of the coral fragments were measured after a 1-h dark acclimation as indicated by the grey shading.

Supplementary Table 1. Species-specific number of nursery corals and reef-based donor colonies acute heat stress tested per site across seasons.

Species	Group	October 2022	March 2023
Acropora florida	Site 1 Donor	12	11
	Site 1 Nursery	12	12
	Site 2 Donor	12	11
	Site 2 Nursery	12	12
Acropora cytherea	Site 1 Donor	14	13
	Site 1 Nursery	13	13
	Site 2 Donor	14	11
	Site 2 Nursery	13	13

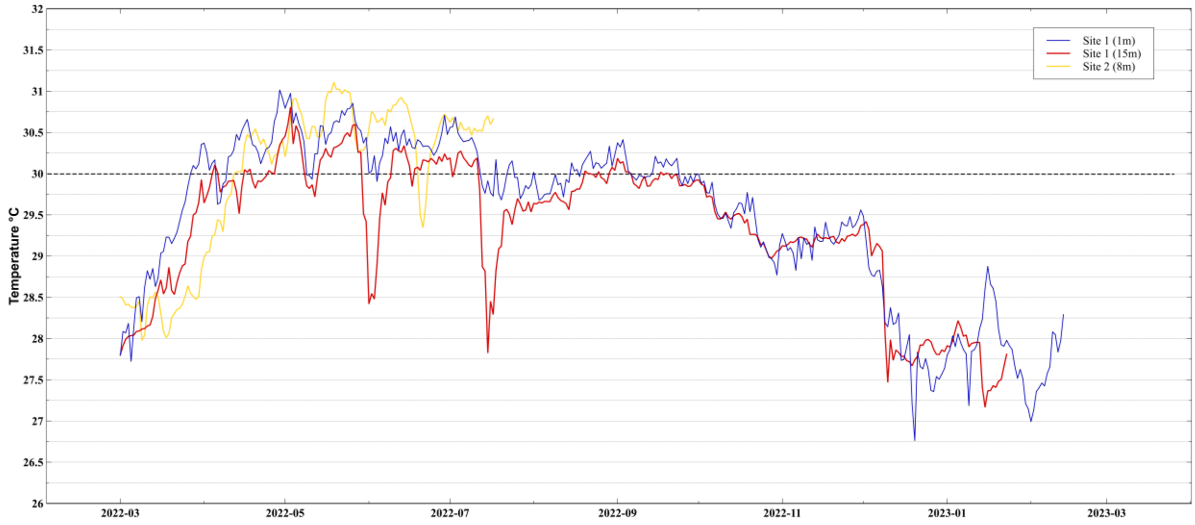

Supplementary Figure 3. In situ temperature data at the common garden nursery (Site 1) and available data for reef Site 2. Temperature data at Site 2 was recorded with HOBO Pendant logger, and temperature at Site 1 by Aqualink Smart Buoy (Sofar Ocean Technologies). The dotted line represents the maximum monthly mean (MMM) based on NOAA Coral Reef Watch climatology (Liu et al., 2014).

Supplementary Table 2. Actual mean maximum treatment temperature of the 3-hour hold phase during acute heat stress assays for each site-condition group across seasons in October 2022 and March 2023 are shown. MMM- maximum monthly mean = 30.52 °C.

**Group**	**MMM**		**MMM+ 4 °C**		**MMM+ 6 °C**		**MMM+ 9 °C**	
Acropora cytherea	October	March	October	March	October	March	October	March
Site 1 Donor	30.75	30.64	34.88	34.82	36.23	36.42	39.63	39.65
Site 1 Nursery	30.75	30.87	34.95	34.92	36.54	36.72	39.75	39.80
Site 2 Donor	30.69	30.91	34.95	34.90	36.59	36.50	39.76	39.73
Site 2 Nursery	31.15	30.88	35.35	34.92	37.04	36.69	40.37	39.72
Acropora florida								
Site 1 Donor	30.88	30.97	34.98	34.87	36.56	36.51	39.81	39.81
Site 1 Nursery	30.64	30.89	35.25	34.79	36.52	36.63	39.91	39.95
Site 2 Donor	30.71	30.78	35.21	34.75	36.52	36.58	39.78	39.72
Site 2 Nursery	30.94	30.94	35.08	35.17	36.84	36.87	40.33	39.92

Supplementary Table 3. Mean maximum temperature differences of the 3h heat-hold phase between acute heat stress assays in October 2022 and March 2023 are shown for each site-condition group. MMM- maximum monthly mean= 30.52 °C.

	Acropora cytherea			
**Group**	**MMM**	**+ 4 °C**	**+ 6 °C**	**+ 9 °C**
Site 1 Donor	-0.11	-0.06	0.19	0.02
Site 1 Nursery	0.12	-0.03	0.18	0.05
Site 2 Donor	0.22	-0.05	-0.09	-0.03
Site 2 Nursery	-0.27	-0.43	-0.35	-0.65
	Acropora florida			
Site 1 Donor	0.09	-0.10	-0.05	-0.01
Site 1 Nursery	0.25	-0.46	0.11	0.04
Site 2 Donor	0.07	-0.46	0.06	-0.06
Site 2 Nursery	0.00	0.09	0.03	-0.41

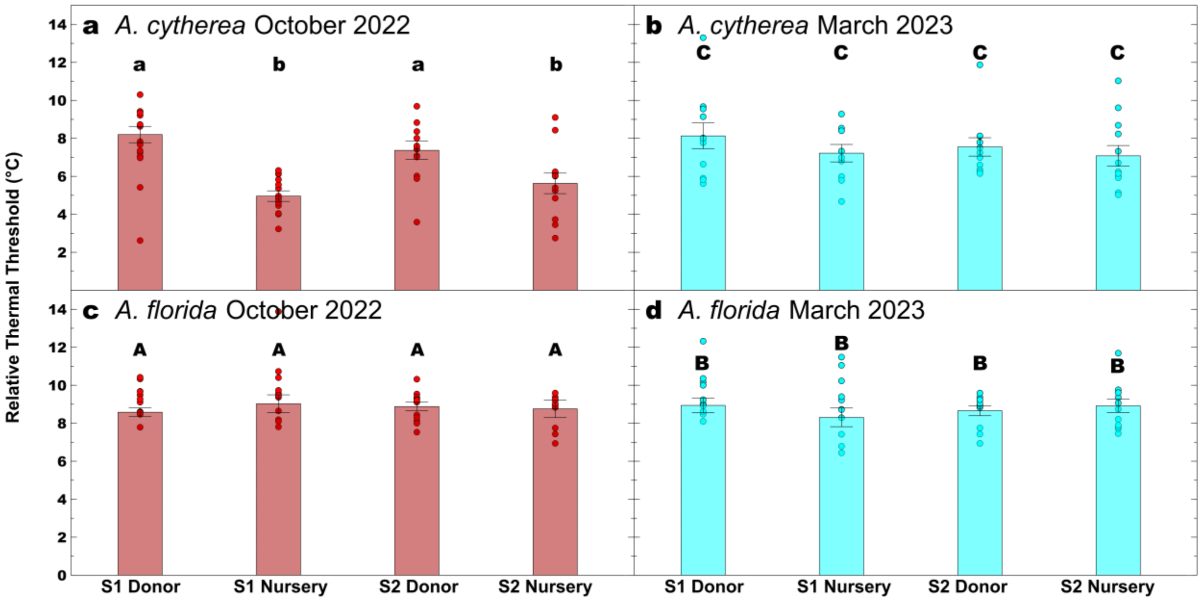

Supplementary Figure 4. Retention and recovery of coral thermal tolerance. Colony-specific thermal thresholds (mean ± S.E.M) relative to the study location's historical maximum monthly mean temperature (MMM=30.52 °C based on in situ interpolation of NOAA's climatology data, Liu et al., 2014) are shown for each site-condition group (i.e., Site 1 Nursery–site, Site 1 – Donor, etc.,) for each season for (a) *Acropora cytherea* in October 2022 and (b) in March 2023; (c) *Acropora florida* in October 2022 and (d) March 2023. Annotated letters highlight significant differences between groups following Tukey HSD multiple comparisons. Letters alternate between small and capital letters for clarity. Dots represent individual data points of the colony replicates.

Supplementary Table 4. Reproducibility of colony-level effective dose 50 (ED50) values across acute heat stress assays (i.e., CBASS) in October 2022 and March 2023. The percent number of corals that re-tested at a difference of < 1 °C (ΔED50 <1 °C) are shown for each species (*Acropora florida* and *Acropora cytherea*) and condition (i.e., Donor and Nursery). n – sample size.

Species	Test	n	ΔED50 <1 °C
A. florida	October 2022 vs March 2023 (Donor)	21	57%
	October 2022 vs March 2023 (Nursery)	21	62%
A. cytherea	October 2022 vs March 2023 (Donor)	24	50%
	October 2022 vs March 2023 (Nursery)	19	37%

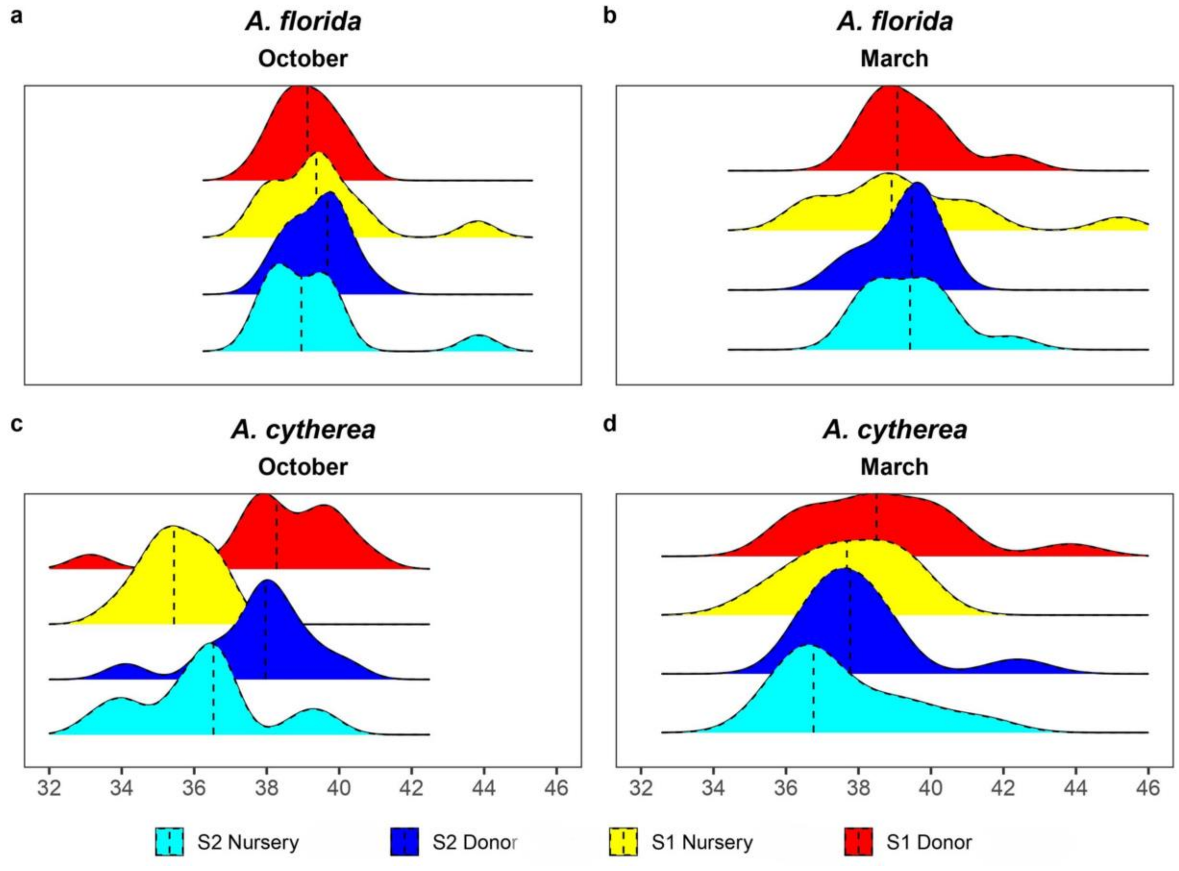

Supplementary Figure 5. Density distribution of effective dose 50 (ED50 °C) derived during acute heat stress assays in October 2022 and March 2023. The dotted line shows the mean ED50 value for each site (i.e., Site 1, Site) and condition (i.e., Nursery, Donor) group for each species (*Acropora florida* and *Acropora cytherea*).

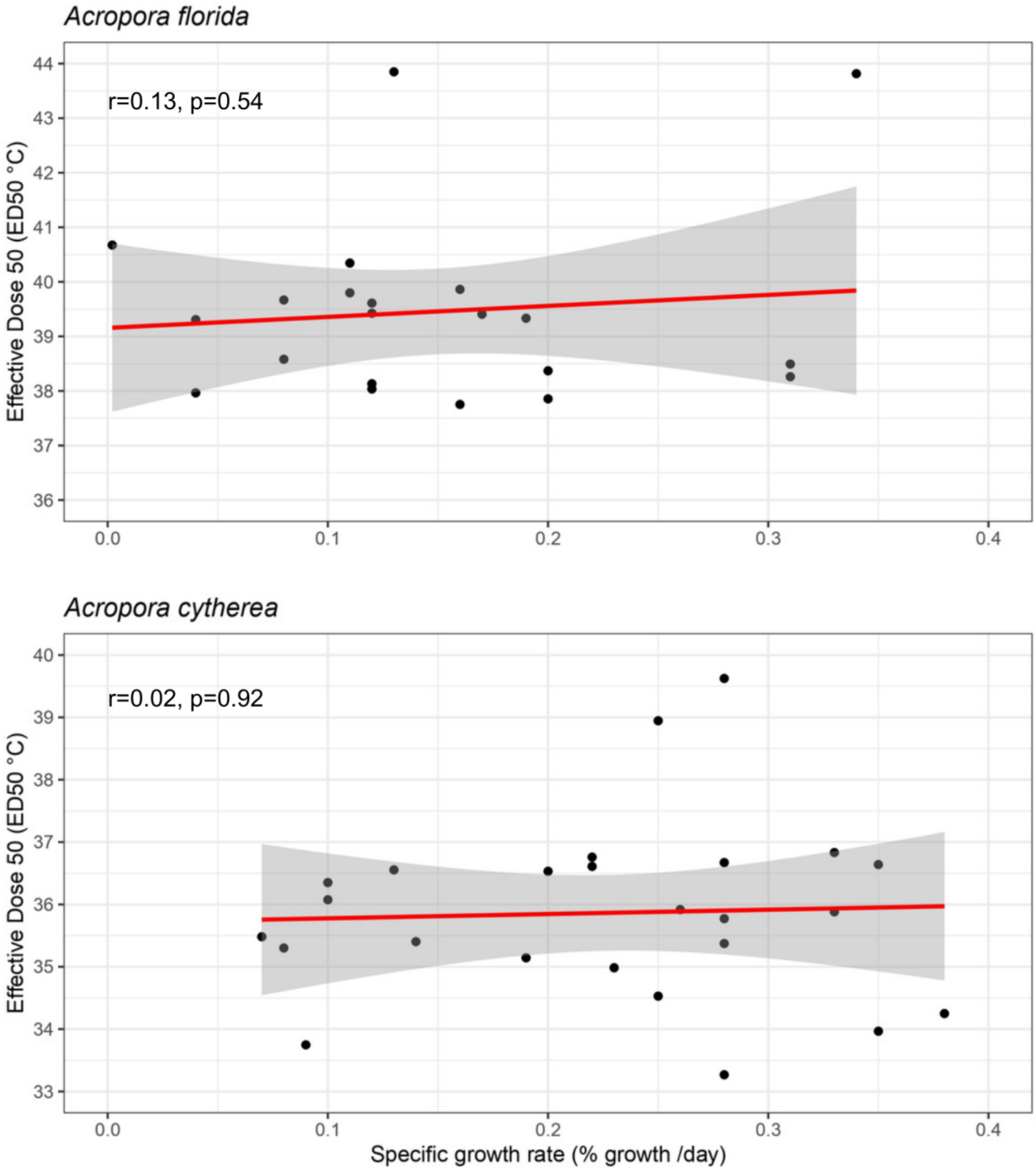

Supplementary Figure 6. Specific growth rates (SGR, % growth/day) plotted against corresponding thermal tolerance thresholds (ED50s) of nursery corals, measured in October 2022 after 176 days of nursery rearing. The Pearson correlation coefficients (r) are shown. Grey-shaded areas represent 95% confidence intervals of the fitted data.
