# Supplementary material for: Species-specific retention vs. recovery of coral thermal tolerance following nursery propagation
Source: Commun Biol. 2025 Aug 28;8:1294. doi: 10.1038/s42003-025-08657-w (PMC12394514; doi:10.1038/s42003-025-08657-w)
Supplement: Supplementary file 3 — Description of Additional Supplementary Materials [file 42003_2025_8657_MOESM3_ESM.pdf]

Description of Additional Supplementary Files

File name: Supplementary Data 1

Description: The source data behind Figures 2-4 and Table 1 in the paper as well as statistical software codes.
