# Supplementary figures and images for: Species-specific retention vs. recovery of coral thermal tolerance following nursery propagation
Source: Commun Biol. 2025 Aug 28;8:1294. doi: 10.1038/s42003-025-08657-w (PMC12394514; doi:10.1038/s42003-025-08657-w)

Mean overlap top 5 vs. bottom 5 ED50s

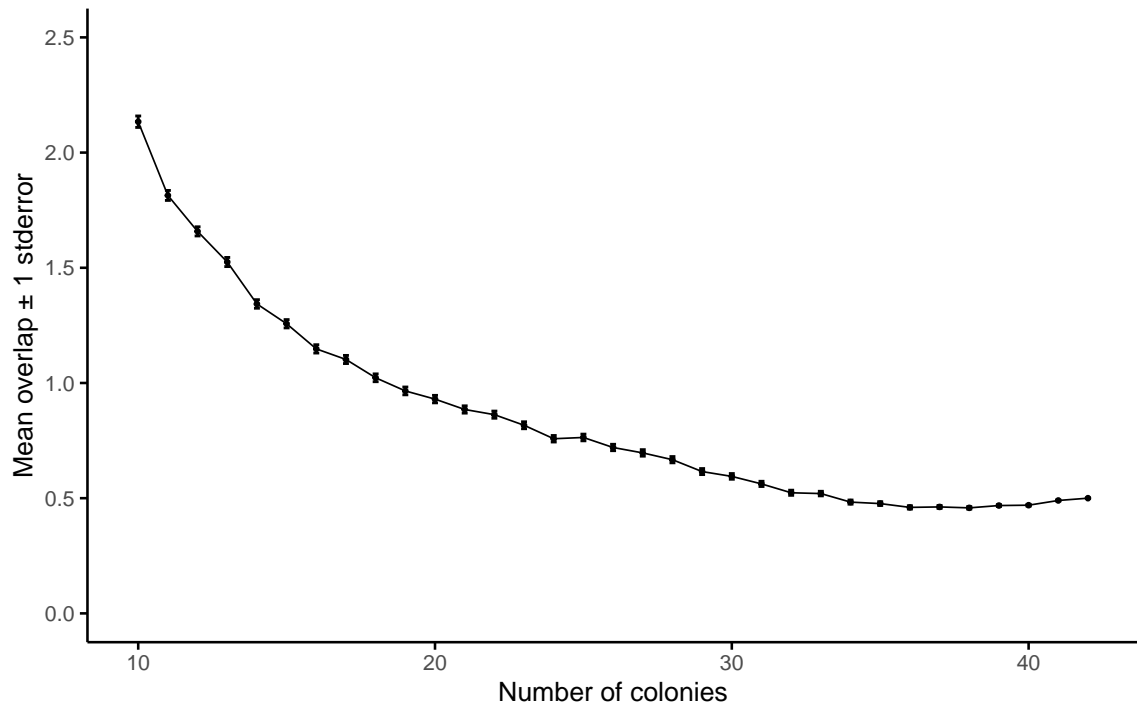

Supplement: Supplementary file 4 — Supplementary Data 1 [file 42003_2025_8657_MOESM4_ESM.zip › Fig4/March2023TopVsBottom.pdf]

Mean overlap top 5 vs. bottom 5 ED50s

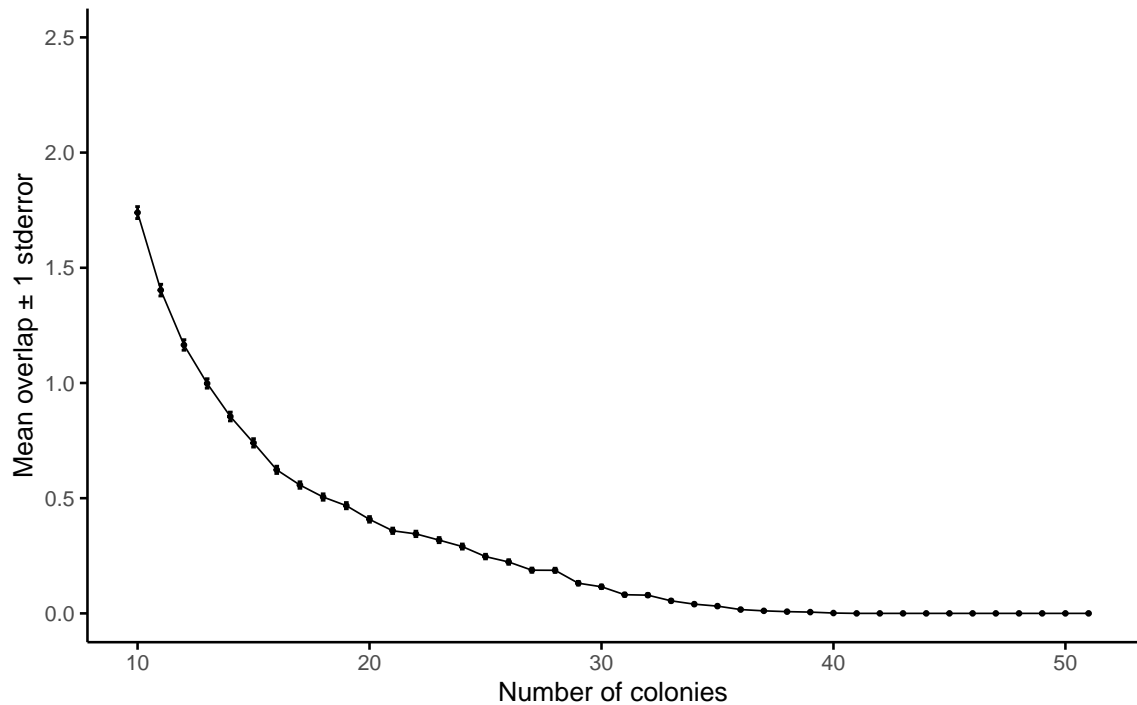

Supplement: Supplementary file 4 — Supplementary Data 1 [file 42003_2025_8657_MOESM4_ESM.zip › Fig4/October2022TopVsBottom.pdf]
