# Supplementary material for: Species-specific retention vs. recovery of coral thermal tolerance following nursery propagation
Source: Commun Biol. 2025 Aug 28;8:1294. doi: 10.1038/s42003-025-08657-w (PMC12394514; doi:10.1038/s42003-025-08657-w)
Supplement: Supplementary file 5 — Reporting Summary [file 42003_2025_8657_MOESM5_ESM.pdf]

nature portfolio

Corresponding author(s): Sebastian Szereday
Last updated by author(s): Mar 27, 2025

Reporting Summary

Nature Portfolio wishes to improve the reproducibility of the work that we publish. This form provides structure for consistency and transparency in reporting. For further information on Nature Portfolio policies, see our Editorial Policies and the Editorial Policy Checklist.

Statistics

For all statistical analyses, confirm that the following items are present in the figure legend, table legend, main text, or Methods section.

n/a	Confirmed	
☐	☒	The exact sample size (*n*) for each experimental group/condition, given as a discrete number and unit of measurement
☐	☒	A statement on whether measurements were taken from distinct samples or whether the same sample was measured repeatedly
☐	☒	The statistical test(s) used AND whether they are one- or two-sided<br>*Only common tests should be described solely by name; describe more complex techniques in the Methods section.*
☐	☒	A description of all covariates tested
☐	☒	A description of any assumptions or corrections, such as tests of normality and adjustment for multiple comparisons
☐	☒	A full description of the statistical parameters including central tendency (e.g. means) or other basic estimates (e.g. regression coefficient) AND variation (e.g. standard deviation) or associated estimates of uncertainty (e.g. confidence intervals)
☐	☒	For null hypothesis testing, the test statistic (e.g. *F*, *t*, *r*) with confidence intervals, effect sizes, degrees of freedom and *P* value noted<br>*Give P values as exact values whenever suitable.*
☒	☐	For Bayesian analysis, information on the choice of priors and Markov chain Monte Carlo settings
☒	☐	For hierarchical and complex designs, identification of the appropriate level for tests and full reporting of outcomes
☐	☒	Estimates of effect sizes (e.g. Cohen's *d*, Pearson's *r*), indicating how they were calculated

Our web collection on statistics for biologists contains articles on many of the points above.

Software and code

Policy information about availability of computer code

Data collection	Neither commercial nor custom code or software was used to collect or generate data. Data collection was purely field (sampling) based.
Data analysis	R Studio and and custom code were used to analyze the data. This custom code was extracted from an available data repository: https://github.com/BarshisLab/CBASS-vs-RSS-Physiology

For manuscripts utilizing custom algorithms or software that are central to the research but not yet described in published literature, software must be made available to editors and reviewers. We strongly encourage code deposition in a community repository (e.g. GitHub). See the Nature Portfolio guidelines for submitting code & software for further information.

Data

Policy information about availability of data

All manuscripts must include a data availability statement. This statement should provide the following information, where applicable:

- Accession codes, unique identifiers, or web links for publicly available datasets
- A description of any restrictions on data availability
- For clinical datasets or third party data, please ensure that the statement adheres to our policy

Data detailed in this study are available at https://github.com/Coralku/commongarden

Research involving human participants, their data, or biological material

Policy information about studies with human participants or human data. See also policy information about sex, gender (identity/presentation), and sexual orientation and race, ethnicity and racism.

Reporting on sex and gender	Not applicable
Reporting on race, ethnicity, or other socially relevant groupings	Not applicable
Population characteristics	Not applicable
Recruitment	Not applicable
Ethics oversight	Not applicable

Note that full information on the approval of the study protocol must also be provided in the manuscript.

Field-specific reporting

Please select the one below that is the best fit for your research. If you are not sure, read the appropriate sections before making your selection.

☐ Life sciences ☐ Behavioural & social sciences ☒ Ecological, evolutionary & environmental sciences

For a reference copy of the document with all sections, see nature.com/documents/nr-reporting-summary-flat.pdf

Ecological, evolutionary & environmental sciences study design

All studies must disclose on these points even when the disclosure is negative.

Study description	Standardized ex situ acute thermal stress assays were used to determine and compare relative thermal tolerance of nursery corals and corresponding reef-based donor colonies to understand whether thermal tolerance of hard corals is lost, retained, and / or recovered following propagation of corals in a coral nursery environment.
Research sample	Two con-generic and foundational coral species were selected for this study to represent coral species that are frequently used in local coral restoration context to support science-based reef restoration. These coral species are important for building the structurally complex reef habitat for coral reef fauna. Corals were tagged and are located in a protected are in Terengganu State, Malaysia. Samples were not manipulated.
Sampling strategy	Reef-based donor colonies were sampled haphazardly at two reef sites along a wide depth range that covered the extent of coral reef substrate at each site, respectively. A minimum distance of 5 meters between con-specific coral colonies was maintained to minimize the possibility of sampling genetic clones. Selected donor colonies were visually healthy, showed no signs of disease, bleaching, or partial mortality. Six months later, donor colonies and corresponding nursery corals were selected randomly for acute heat stress testing. For each species, a total of 14 corals per group were selected. A total of four groups per species were tested: donor colony reef site 1 and 2, and corresponding nursery corals originating from donor colonies from reef site 1 and 2. The chosen sample size of 14 is statistically reasonable based on power calculations, confidence interval considerations, and feasibility constraints. It ensures a good balance between precision and practicality while still allowing for a robust estimate of the population mean. Furthermore, for moderate effect sizes, the Central Limit Theorem (CLT) suggests that $n \geq 10\text{-}15$ is often sufficient for approximating a normal distribution of the sample mean. Practically, due to the wide sampling range across depth at each reef site, the coral sample size reflects a representative sample of the site-specific population for each species (since the reef sites are rather small in terms of areal extent). the possibility of sampling genetic clones.
Data collection	Data were collected by Sebastian Szereday and Chew Kok Lynn. For quantitative comparison of species-specific standardized thermal tolerance thresholds between nursery corals and reef-based donors, photochemical yields (Fv/Fm) were measured by recording the dark-acclimated maximum photosynthetic efficiency of PSII of the coral algae. Measurements were taken by pulse amplitude modulated (PAM) fluorometry to derive a physiological measure reflective of heat stress tolerance of corals. These Measurements were recorded 7 hours after the start of short-term acute heat stress assays which followed a standardized experimental design (i.e., CBASS). During the last 30 minutes of the assays, corals were kept in complete darkness for 30 minutes to ensure dark acclimation. These assays were repeated six months in March 2023 later for every coral tested in October 2022.
Timing and spatial scale	Thermal stress assays were conducted in October 2022 between the 8th and 15th of October. Thermal stress assays were repeated between between the 13th and 20th of March 2023. These periods were deliberately chosen to account for local seasonal variability in ocean conditions, specifically water temperature, wind, waves, and storm activity. This sampling frequency thus enabled to test whether thermal tolerance changes across local seasons over the course of 1 year. The year period would be of relevance to coral restoration practitioners.
Data exclusions	Three measurements of photochemical yields (Fv/Fm) were taken after each acute heat stress assay for each coral measured across three locations along the corals surface. The standard deviation was calculated for each temperature treatment and species-specific group (i.e., donor colony reef site 1, donor colony reef site 2, nursery corals reef site 1, and nursery corals reef site 2). Next, the

difference between the highest and lowest measurement for each coral in each treatment was calculated. If this difference exceeded the standard deviation, the highest measurement was excluded. The lowest measurement was only excluded when the difference of other two (higher) measurements were within the standard deviation. If the exclusion of neither the highest nor the lowest of three measurements resulted in the difference between two measurements to be less than the standard deviation, the two highest measurements were excluded to ensure a conservative measurement approach for later modelling of thermal tolerance. Therefore, this approach ensured a conservative exclusion of high Fv/Fm measurements above the group-specific and treatment-specific standard deviation, while reducing biases due to low measurement outliers. Furthermore, minimal data exclusion occurred during pairwise comparison of thermal tolerance of reef-based donor colonies vs nursery coral. The reason for this exclusion is the mortality of either the nursery coral or the donor colony, preventing pairwise testing. No further data were excluded from the study.

Reproducibility

Thermal stress assays followed a standardized protocol using the Coral Bleaching Automated Stress System (CBASSS). This system was designed to ensure standardized replication of acute short-term thermal stress assays.

Randomization

Reef-based donor colonies were haphazardly selected at two reef sites between a depth range of 4.4 - 15.2 m water depth, each at a minimum distance of 5 m to each other. This minimized the possibility of sampling genetic clones. During CBASS assays, the position of each coral fragment within each CBASS treatment tank was randomized using a random number generator. Further randomization was not relevant to this study.

Blinding

This study investigated thermal tolerance of reef-based donor colonies vs corresponding nursery offspring. Since thermal tolerance and other relevant attributes of the coral colony were entirely unknown during the selection of corals for the experiments, blinding was not relevant for this study.

Did the study involve field work? [x] Yes [ ] No

Field work, collection and transport

Field conditions

Average water temperature are detailed in the electronic supplementary material and should be accessed there for proper conceptualization of field conditions.

Location

We conducted a year-long investigation to assess coral thermal tolerance dynamics at an ongoing restoration site around Pulau Lang Tengah (5°47’43.2"N, 102°53’39.7"E), in northeastern Peninsular Malaysia.

Access & import/export

The research was conducted under permit number Prk. ML. 630-7Jld.12 (90), issued by the Department of Fisheries (DoF) Malaysia (Jabatan Perikanan Malaysia), and permit number EPU 40/200/19/3711 (13) issued by the Economic Planning Unit (Unit Perancang Ekonomi). No biological material was extracted from the study location.

Disturbance

Coral biomass was removed from donor colonies. To ensure minimal sampling impact on the coral, only 2-3 cm large coral pieces were extracted to extract less than 1% of the corals total biomass.

Reporting for specific materials, systems and methods

We require information from authors about some types of materials, experimental systems and methods used in many studies. Here, indicate whether each material, system or method listed is relevant to your study. If you are not sure if a list item applies to your research, read the appropriate section before selecting a response.

Materials & experimental systems

n/a	Involved in the study	
[ ]	[ ]	Antibodies
[ ]	[ ]	Eukaryotic cell lines
[ ]	[ ]	Palaeontology and archaeology
[ ]	[x]	Animals and other organisms
[ ]	[ ]	Clinical data
[ ]	[ ]	Dual use research of concern
[ ]	[ ]	Plants

Methods

n/a	Involved in the study	
[x]	[ ]	ChIP-seq
[x]	[ ]	Flow cytometry
[x]	[ ]	MRI-based neuroimaging

Antibodies

Antibodies used

Not applicable

Validation

Not applicable

Eukaryotic cell lines

Policy information about cell lines and Sex and Gender in Research

Cell line source(s)	Not applicable
Authentication	Not applicable
Mycoplasma contamination	Not applicable
Commonly misidentified lines (See ICLAC register)	Not applicable

Palaeontology and Archaeology

Specimen provenance	Not applicable
Specimen deposition	Not applicable
Dating methods	Not applicable

☐ Tick this box to confirm that the raw and calibrated dates are available in the paper or in Supplementary Information.

Ethics oversight	*Identify the organization(s) that approved or provided guidance on the study protocol, OR state that no ethical approval or guidance was required and explain why not.*

Note that full information on the approval of the study protocol must also be provided in the manuscript.

Animals and other research organisms

Policy information about studies involving animals; ARRIVE guidelines recommended for reporting animal research, and Sex and Gender in Research

Laboratory animals	Not applicable
Wild animals	Not applicable
Reporting on sex	Not applicable
Field-collected samples	The study involved samples from the field. However, the conducted experiments lasted only for 18 hours in the field and (surviving) samples were returned immediately after the experiment to the source location.
Ethics oversight	No ethical approval was required as per local governmental regulations. A valid research permit was required to carry out the sampling, as stated above.

Note that full information on the approval of the study protocol must also be provided in the manuscript.

Clinical data

Policy information about clinical studies

All manuscripts should comply with the ICMJE guidelines for publication of clinical research and a completed CONSORT checklist must be included with all submissions.

Clinical trial registration	Not applicable
Study protocol	Not applicable
Data collection	Not applicable
Outcomes	Not applicable

Dual use research of concern

Policy information about dual use research of concern

Hazards

Could the accidental, deliberate or reckless misuse of agents or technologies generated in the work, or the application of information presented in the manuscript, pose a threat to:

No	Yes	
☒	☐	Public health
☒	☐	National security
☒	☐	Crops and/or livestock
☒	☐	Ecosystems
☒	☐	Any other significant area

Experiments of concern

Does the work involve any of these experiments of concern:

No	Yes	
☒	☐	Demonstrate how to render a vaccine ineffective
☒	☐	Confer resistance to therapeutically useful antibiotics or antiviral agents
☒	☐	Enhance the virulence of a pathogen or render a nonpathogen virulent
☒	☐	Increase transmissibility of a pathogen
☒	☐	Alter the host range of a pathogen
☒	☐	Enable evasion of diagnostic/detection modalities
☒	☐	Enable the weaponization of a biological agent or toxin
☒	☐	Any other potentially harmful combination of experiments and agents

Plants

Seed stocks	Not applicable
Novel plant genotypes	Not applicable
Authentication	Not applicable
